# Quantifying grating defects in X-ray Talbot-Lau interferometry through a comparative study of two fabrication techniques

**DOI:** 10.1038/s41598-025-98148-z

**Published:** 2025-04-24

**Authors:** Alexandre Pereira, Simon Spindler, Zhitian Shi, Lucia Romano, Michał Rawlik, Federica Marone, Daniel Josell, Martin Stauber, Marco Stampanoni

**Affiliations:** 1 Institute for Biomedical Engineering, ETH Zürich and University of Zürich, Zürich, Switzerland; 2 Swiss Light Source, Paul Scherrer Institute, Villigen, Switzerland; 3 Centre for Photonic Science Systems, Electrical Engineering Division, Department of Engineering, University of Cambridge, Cambridge, UK; 4 Materials Science and Engineering Division, NIST, Maryland, USA; 5 GratXray, 5234 Villigen, Switzerland

**Keywords:** Materials for optics, X-rays, Optics and photonics, X-ray tomography, Biomedical engineering

## Abstract

The performance of an X-ray grating interferometry system depends on the geometry and quality of the gratings. Fabrication of micrometer-pitch high-aspect-ratio gold gratings, which are essential for measuring small refraction angles at higher energies, is challenging. The two widely used technologies for manufacturing gratings are based on gold electroplating in polymeric or silicon templates. Here, gratings manufactured by both approaches were inspected using conventional microscopy, X-ray synchrotron radiography, and computed laminography to extract characteristic features of the gratings profile to be modeled accurately. These models were used in a wave-propagation simulation to predict the effects of the gratings’ geometry and defects on the quality of a Talbot-Lau interferometer in terms of visibility and absorption capabilities. The simulated outcomes of grating features produced with both techniques could eventually be observed and evaluated in a table-top Talbot-Lau-Interferometer.

## Introduction

X-ray imaging is a widely used technique in medical, industrial, and scientific fields. However, conventional X-ray imaging relies on the difference in attenuating power of the sample when X-rays pass through it, which gives limited contrast in objects with similar attenuation coefficients, such as soft tissue in medical imaging. In contrast, X-ray phase contrast imaging is a category of methods sensitive to the refraction rather than the absorption of X-rays as they pass through the sample. Coherent refraction, on features that can be spatially resolved, enhances image quality in low-density materials, particularly at high resolution. Stochastic refraction on multiple interfaces, the so-called dark field, has been shown to provide high diagnostic potential in various fields, such as breast, bone, and lung imaging^1–6^.

X-ray grating interferometry (XGI) is one of the methods sensitive to refraction^7–9^. Because X-ray detectors only measure the intensity of the beam, which is directly linked to the beam attenuation, the coherent and stochastic refraction is measured in XGI utilizing a set of high-aspect ratio (HAR) gratings to modulate the signal into an intensity variation^10,11^. A Talbot-Lau interferometer^8,12^ is an XGI that uses three gratings: G0, G1 and G2. A phase-shifting diffraction grating G1 induces an intensity pattern of parallel lines, with a period of several micrometers, in the plane of G2. G2 is a periodically-opaque analyzer grating creating a low-frequency Moiré pattern, which is then resolved by the detector behind it. Because the effect only occurs if the X-ray source is sufficiently small, a second periodically-opaque (absorption) grating, G0, is placed in front of a high-power, large-spot source to create an array of narrow sources. For appropriate combinations of the grating separations and pitches these narrow sources can be made to produce parallel-line patterns that coincide in the G2 plane, thereby maintaining and reinforcing the Moiré pattern.

The complex index of refraction of a material is expressed as $n = 1 - \delta + i \beta$ where $\delta$ and $\beta$ describe the shift in phase and the attenuation of the beam, respectively. The complex refractive index imposes a phase shift on the wavefront when propagating through an object, with the gradient of the phase shift leading to a refraction of the beam by an angle $\alpha$, which is directly related to the retrieved phase shift $\phi$ of the phase stepping curve by^7^:

1 $$\begin{aligned} \alpha = \frac{p_2}{2 \pi d} \phi . \end{aligned}$$

Here, $p_2$ is the pitch of G2, and *d* is the distance between the sample and G2. Therefore, the angular sensitivity in a Talbot-Lau interferometer increases with smaller grating pitches and longer distances between the sample and G2, as smaller refraction angles can be measured, which lead to bigger intensity changes on the detector^13^. Similarly, uncertainty in the measured signal $\sigma _{\phi }$ propagates to uncertainty in the evaluated refraction angle using:

2 $$\begin{aligned} \sigma _{\alpha } = \frac{p_2}{2 \pi d} \sigma _{\phi }. \end{aligned}$$

The uncertainty $\sigma _{\phi }$ for a photon-counting detector (absent a sample) is directly linked to^14,15^:

3 $$\begin{aligned} \sigma _{\phi } \propto \frac{\sqrt{2}}{V \sqrt{I}}, \end{aligned}$$

where *V* is the visibility (the contrast of the Moiré fringe measured on the detector behind G2) and *I* is the number of detected photons. Both *V* and *I* depend on the local quality of the gratings, including the geometry of the microstructures, the materials, and defects.

The design of an XGI system depends on several parameters that mutually influence the uncertainty of the measured refraction angle. Generally, small grating periods are targeted, as systems often have constraints on their total length. However, thicker gratings with taller Au-filled features are needed to efficiently block incoming X-rays, especially at higher X-ray energies. With insufficient height of the absorbing Au, the interferometer’s visibility is reduced. That said, as the aspect ratio of the features increases with increasing grating thickness and decreasing pitch, defects are more likely, leading to reduced visibility, heterogeneous visibility distributions, or unnecessary absorption of incoming photons that do not contribute to the interference pattern. The grating manufacturing technique determines the range of achievable aspect ratios. The two primary manufacturing processes for gold X-ray gratings used in grating interferometry utilize: Au-electroplating through an insulating polymeric template on an electrically conductive layer, as in Deep X-ray lithography and LIGA (Lithography, Galvanoforming, and Plastic Molding) (X-LIGA)^10,16,17^, or fabrication of an etched silicon template, such as by deep reactive ion etching (DRIE) of silicon^18^, followed by a bottom-up Au electroplating^19^. A more detailed description of the techniques is reported in the next section.

In this study, two pairs of grating sets were compared; the first set contained two X-LIGA gratings (MicroWorks GmbH), the second set contained two in-house manufactured DRIE and Au bottom-up filled gratings. All the gratings have a pitch of 4.2 µm. The X-LIGA gratings have a height of approximately 180 µm, while the DRIE gratings have a height of approximately 145 µm and 153 µm for G0 and G2, respectively. Both X-LIGA and DRIE gratings have comparatively widely spaced transverse line segments (bridges) to stabilize the high-aspect-ratio lamellae during the wet processes (development and electroplating for X-LIGA, electroplating for Au bottom-up of DRIE gratings). This work aims to analyze and understand the effects of the individual geometries and defects of the two grating fabrication techniques on the sensitivity of imaging systems incorporating them, defined as the inverse of Eq. 3 as $S = \sqrt{I}V$. Previous work has shown the ability to obtain preliminary information about height and lamella inclination in individual gratings by angular X-ray transmission imaging.^20^. Here, an investigation of the limitations of these fabrication techniques and, eventually, their influence on an XGI system is presented. The work begins by analyzing the transmission profile of gratings produced by DRIE and X-LIGA using synchrotron imaging and studying their cross-sectional profile using scanning electron microscopy (SEM) and computed laminography (CL), respectively. This information is used to model the individual defects and predict their influence using wave propagation simulations. Finally, the gratings are mounted on a table-top Talbot-Lau interferometer and compared pairwise.

## Grating fabrication techniques

In the context of X-ray gratings fabrication, the process of LIGA involves using lithography with exposure light in the X-ray wavelength to pattern a thick photoresist layer on an electrically conducting substrate. After development, the patterned photoresist is then electroplated with a high-Z metal, such as gold or nickel. X-LIGA is a highly precise and versatile manufacturing process, allowing for the fabrication of gratings with a wide range of geometries and periods. The electroplating process uses a seed layer deposited on the substrate before the photoresist; this allows the use of a wide variety of substrates, even soft and flexible materials, such as graphite, that permit bending of the grating after the molding and electroplating process^21^. Such bending is usually required to match the cone beam geometry of an XGI system so that the gratings lamellae are aligned to the X-rays across the entire area of the grating. X-LIGA enables the highly precise manufacture of HAR microstructures with large structural thicknesses ranging from hundreds to thousands of micrometers^16^ and line width in the range of few to tens of micrometers. The X-LIGA grating of this study has an aspect ratio of 85:1 with a pitch size of 4.2 µm, aspect ratios of 90:1 for pitch 4.8 µm^16,22^ are reported as state-of-the-art for this technique. In contrast, silicon etching can be realized by chemical^23^ or plasma-assisted process^18^ to selectively remove material from a silicon substrate, creating high-precision microstructures. X-ray gratings typically involve lithography to pattern a photoresist layer on a silicon wafer, the pattern being subsequently transferred to the underlying material. In DRIE, high aspect ratio vertical microstructures are realized in the silicon using the so-called Bosch process^18,24–28^. DRIE can realize fan-shaped gratings to avoid subsequent bending of the substrate to align the grating lamellae with the X-rays^25,27^. The conventional process of DRIE has aspect ratio in the range of 50:1^29^, higher AR in the range of 100:1 are reported for cryo-etching usually for submicrometer pitch size^29^, new silicon etching methods such as metal-assisted chemical etching can achieve aspect ratio in the range of 1000:1 and even higher^23^. In this study, we optimized a conventional Bosch process for DRIE^18^ for a grating with pitch size of 4.2 µm and aspect ratio in the range of 70:1. After DRIE, the silicon template is conformally coated with a conductive seed layer deposited by atomic layer deposition (ALD) for the subsequent Au electrodeposition. Usually, a thin layer of Al_2_O_3_ is used as an adhesion layer for a thin layer of Pt. In a gold-sulfite electrolyte, the Au electrodeposition is realized using a Bismuth-stimulated bottom-up modality^19^ to obtain a dense void-free Au filling of the Si trenches, including in gratings bent to yield intrinsic curvature after Au-filling for improved imaging^30^. The combination of DRIE and Au bottom-up electroplating offers a robust high precision fabrication method and good material compatibility with X-rays, making it a popular choice for XGI^31–33^. For simplicity, these gratings are addressed as DRIE gratings in the following text.

The grating features, such as pitch size and duty cycle, i.e., the ratio between the width of the transmitting line and the pitch (schematically shown in Fig. 2a), are mainly determined by the patterning of the thick photoresist layer (exposure and development) in X-LIGA. In DRIE, the pattern transfer in the hard mask and the silicon etching process are mainly responsible for the grating features. In both methods, X-LIGA and DRIE, the electroplating process controls the height and the density of the Au lamellae. Both methods can produce high-precision HAR gratings with micrometer periods, making them well-suited for X-ray phase contrast imaging using XGI.

## Inspection of individual gratings

### X-ray radiography imaging of DRIE and X-LIGA gratings

**Fig. 1:**
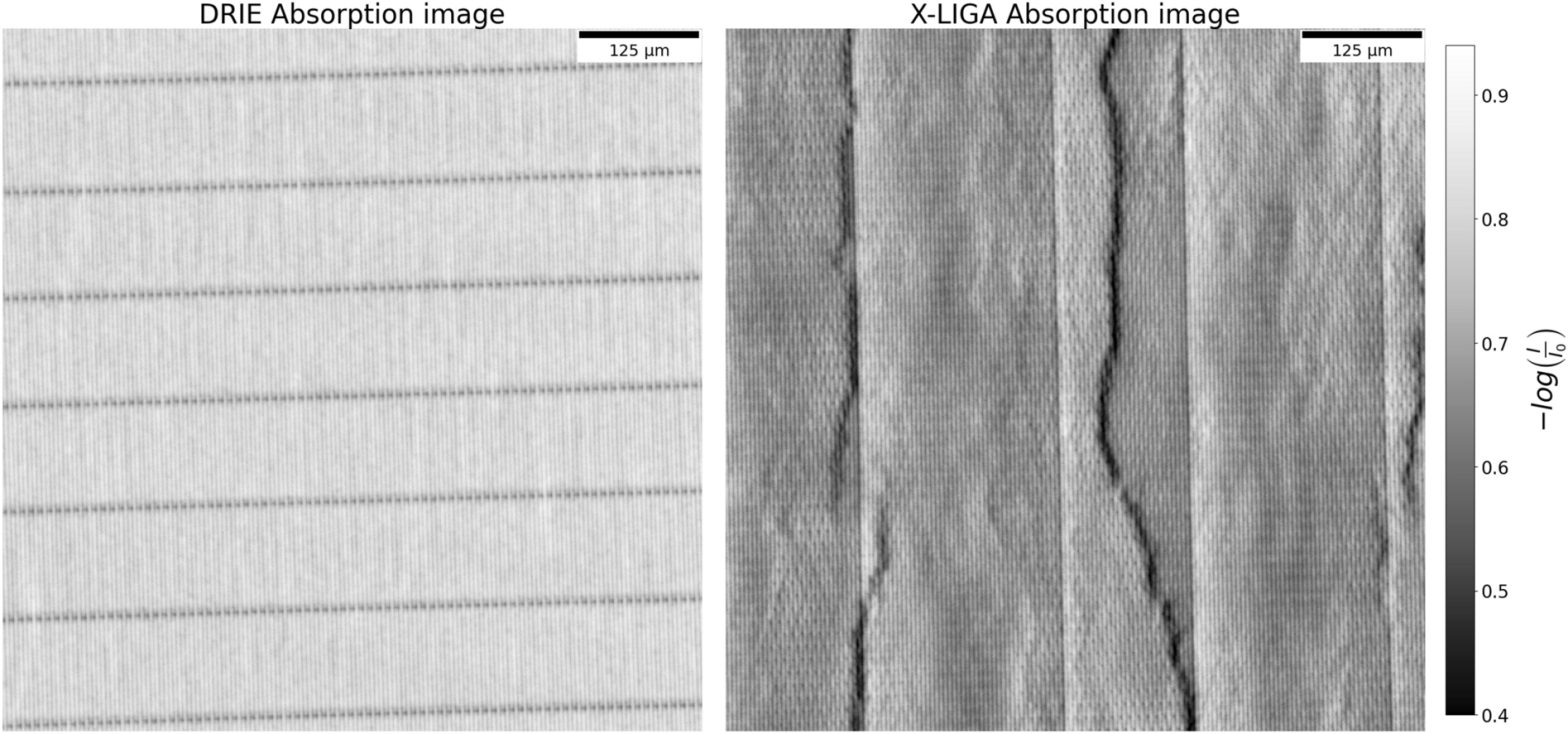
Absorption images of a DRIE and an X-LIGA grating obtained with a monochromatic beam of 45 keV at the TOMCAT beamline. The DRIE grating exhibits an essentially period pattern indicating uniform and dense Au filling. The dark features arise from the bridges in the silicon template. The X-LIGA grating shows irregularities depicting areas with lower absorption that propagate through the grating. The colorbar values indicate the negative logarithm of the ratio in intensity with and without the grating in the beam (i.e., absorption in gold yielding higher values). Higher average absorption in the DRIE gratings, despite shallower grating height, arises from a decrease in the effective duty cycle due to the trapezoidal trench shape.

In order to obtain initial information about the quality and uniformity of the gratings, high-resolution X-ray images were obtained at the TOMCAT (TOmographic Microscopy and Coherent rAdiology experimenTs) beamline of the Swiss Light Source. Fig. 1 shows the absorption images of a grating produced by DRIE and X-LIGA. The DRIE grating absorbs more than the X-LIGA grating despite having shallower trenches (145 µm vs. 180 µm). The absorption image exhibits uniform oscillations aside from higher transmission where the bridges are located. The X-LIGA grating, in contrast, exhibits substantial irregularities, including areas with high transmission whose shapes do not correspond to the lamellae of the grating. The effect of the different bridge fractions in the gratings: 1 % in DRIE, 10 % in X-LIGA, is also evident. The transmission images provide significant insight into the quality of the gratings.

**Fig. 2:**
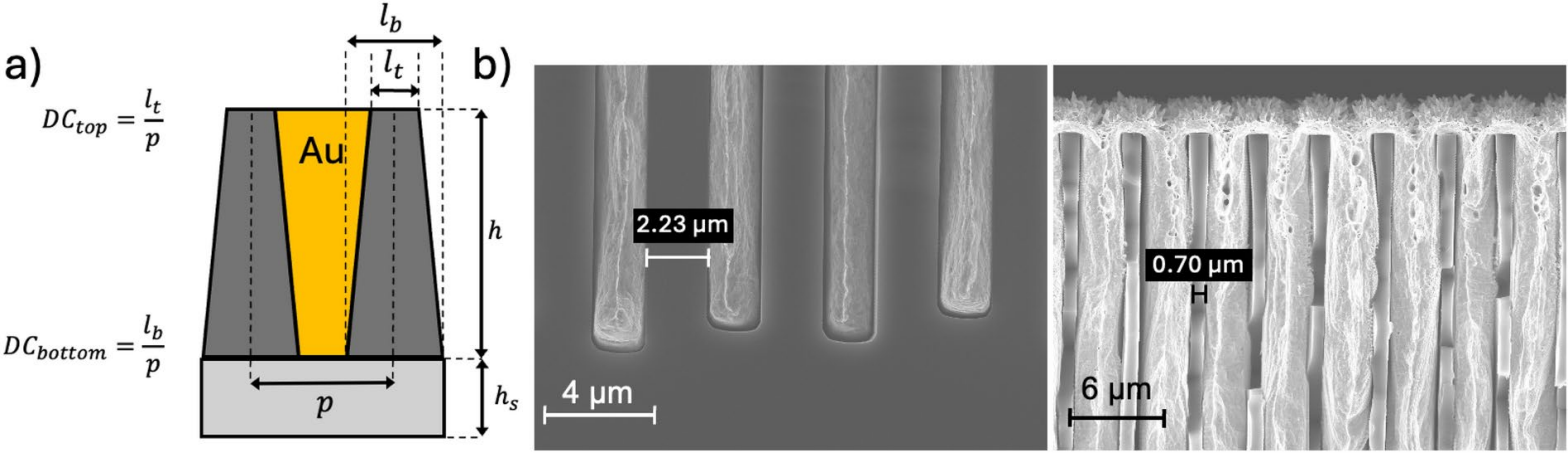
(**a**) Schematic of a grating geometry. The duty cycle is defined as the ratio between the transmitting linewidth and pitch. Additionally, to incorporate tapering geometry, two duty cycles for the top and bottom area are defined. *h* and $h_s$ indicate the height of the grating and the height of the substrate, respectively. (**b**) SEM cross-sectional images of an Au-filled DRIE grating with 153 µm height. The grating shows uniform gold filling with minor voids at the top. Additionally, the grating has substantial tapering, i.e., different duty cycles at the top and bottom of the grating.

### DRIE: scanning electron microscopy

Although the DRIE gratings have shallower trenches, they absorb more incoming X-rays. To understand this effect, a cross-sectional profile after gold electroplating by SEM was acquired. The cross-section was realized by simply cleaving the Si wafer along the $<100>$ crystal orientation. The electrodeposited Au has a microcrystalline structure (face-centered cubic), thus, it has deformed plastically during the cleaving, yielding typical cup and cone features on the fracture surface seen by SEM in Fig. 2b. Filling has occurred in a generally bottom-up manner, the deposit being fully dense and crystalline aside from some voids at the top of the trenches where a rough (passive) deposit is observed. A comparison of the images of the trench tops and bottoms makes clear that the grating has tapering features, i.e., different duty cycles at the bottom and top, leading to a trapezoidal shape of the trenches. The tapered shape is typical of the Bosch process in high-aspect-ratio micro-structures (here, the aspect ratio exceeds 70:1). X-ray absorption in the Au-filled trapezoidal profile is higher than that of a rectangular grating with an equal average duty cycle. This is because substantial absorption occurs across a larger fraction of the pattern, given the exponential dependence of absorption on the thickness of the transited Au. As a result, X-rays propagating through the grating experience a smaller fraction of the gold-free area than in a rectangular grating. Additionally, voids in the deposit decrease the average density in the uppermost 30 µm grating height in G0 (not shown) and 5 µm in G2 (Fig. 2b); the effective Au thickness in the DRIE gratings is thereby reduced somewhat below the already shallower trench height. An estimation of the effective Au thickness is discussed later.

### X-LIGA: computed laminography

Cross-sectional images of X-LIGA gratings have been reported for low aspect ratio gratings^10^ and are usually produced at the border of the pattern since it is not possible to cleave the polymeric template like the crystalline silicon grating. A proper sectioning implies a mechanical dicing of the Au lamellas requiring a consequent polishing, which is challenging due to the very different ion milling rates of Au and polymer components of the grating.

A method specifically designed for imaging planar objects is computed laminography (CL)^34^. In contrast to standard computed tomography (CT), the rotation axis in CL is tilted with respect to the beam path to mitigate the strong absorption of the sample parallel to the lateral elongation^35^. It has been demonstrated that CL produces volumes with fewer artifacts and better isotropic resolution compared to CT^34^.

**Fig. 3:**
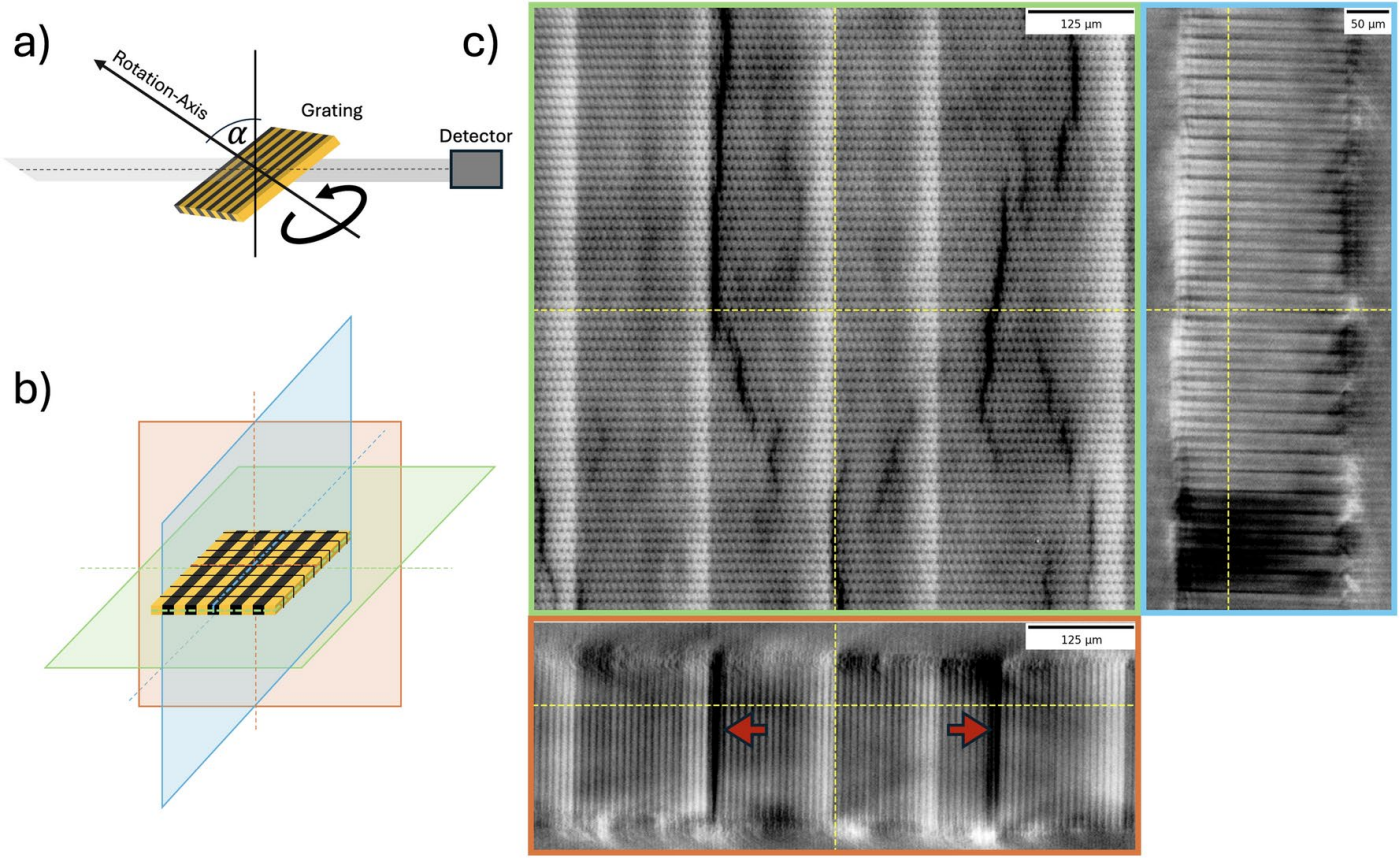
(**a**) Schematic of the acquisition setup of the laminography scan. The rotation axis is tilted in the beam direction by 30 °. The volume was acquired at the TOMCAT beamline with 6000 projections over 360 degrees. (**b**) Schematic of the reconstruction volume with the colors visualizing the different planes in the reconstructed volume. (**c**) Reconstructed volume of an X-LIGA grating with 4.2 µm pitch and 180 µm height; the frames around each image correspond to the planar view indicated schematically in (**b**). The red arrows highlight areas in the grating that are highly transmissive and propagate through the full thickness of the grating. In the orange plane, the periodicity of the bridges of the gratings is depicted. A comparison of the laminography to an optical microscope image of the grating’s surface is shown in the supplementary material (see Fig. S1), additionally demonstrating that the areas propagate through the full grating height.

Fig. 3 depicts three planar views of the reconstructed volume. The darker regions in the reconstructed volumes indicate lower absorbing materials, which resemble the contrast differences seen in the absorption image (Fig. 1 X-LIGA). Those lines propagate through the grating, indicated by the red arrows. Artifacts are observed at the grating interfaces with the air and the substrate due to the limited sampling of the frequency space, which is typical for CL and increases with a larger tilt angle^36^.

## Wave-propagation simulation

### Influence of tapering on the sensitivity

Generally, the properties of gratings impact the sensitivity of XGI systems in a non-trivial manner. Although particular cases can be evaluated, a broad approach utilizing wave-propagation simulation (*RaveSim* package^37^) was used to investigate the individual properties of the grating parameters on the sensitivity.

**Fig. 4:**
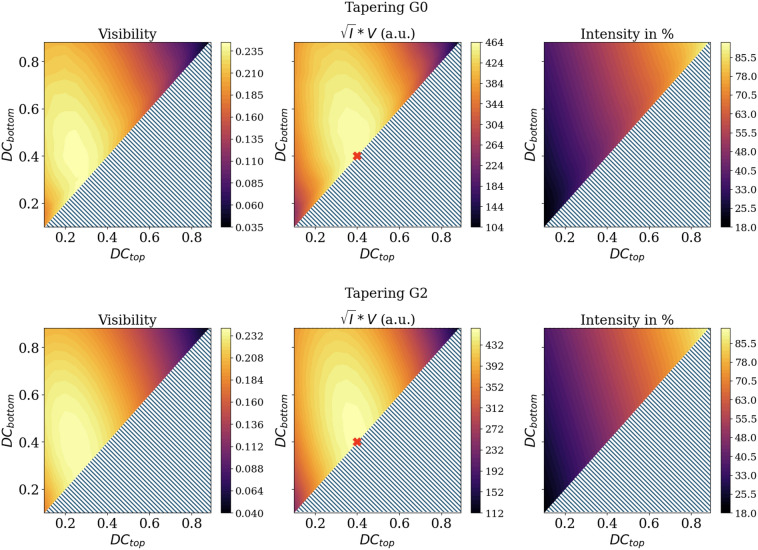
Effect of tapering of G0 or G2, as indicated, on the visibility, sensitivity, and transmission. The grating is modeled as a trapezoidal shape where the top duty cycle is smaller than or equal to the bottom duty cycle. The other grating in each simulation is modeled as a rectangular X-LIGA grating with a height of 180 µm and a duty cycle of 50 %. The intensity is expressed relative to that from a pure silicon grating. The highest sensitivity is reached at a duty cycle of 40 % top and bottom, marked by the red *x*, for both scenarios.

In the first step, the influence of tapering on the sensitivity is explored by varying the top and bottom duty cycle of the gratings, i.e., modeling the gratings with a trapezoidal shape, for G0 and G2 with a height of 140 µm, while leaving one of the gratings in each configuration as an X-LIGA grating with 180 µm height and 50 % duty cycle. The simulation meets the specification of the XGI as used in the experimental investigation, explained later (see Fig. 9a). The duty cycle was incremented by a step size of 5 %, starting from 10 to 90 % for bottom and top duty cycle. In Fig. 4, it is observed that tapering can increase the visibility, and an optimum in visibility can be found for duty cycles at the trench top and bottom ($DC_{top}-DC_{bottom}$) of $25- 35\,\%$, respectively, for the trapezoidal grating used as G0 and of $25- 40\,\%$ for G2. However, due to the substantial decrease in transmission that accompanies non-rectangular trench shapes, the optimum sensitivity is at 40–40% (i.e., rectangular) for use as either G0 or G2.

### Effect of local trench defects on sensitivity

**Fig. 5:**
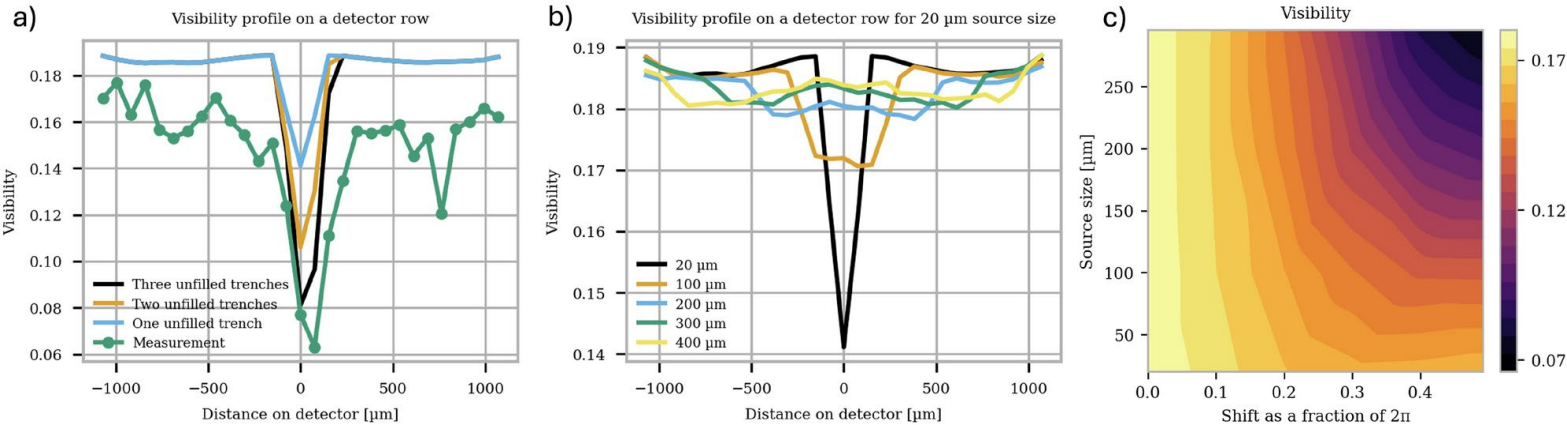
(**a**) Effect on the visibility assessed at the detector of trenches missing absorbing Au (i.e., unfilled) in G0 for a constant source size of 20 µm on a detector row. The more trenches are missing, the larger the drop in visibility. The drop extends to the neighboring pixel to the right of the center with increasing missing trenches, as the modeled grating is asymmetric around the source, with more missing trenches on the right. (**b**) Effect of a single missing absorbing trench for different source sizes. While the decrease of visibility is largest for pixel(s) in-line with the unfilled trench(es) for a small source size, visibility is reduced in pixels at larger distances from those in-line with the unfilled trench(es) for larger source sizes. While the drop is stronger for a small source size, visibility reduces for larger source sizes over a larger area. (**c**) Influence of different period shifts on the visibility for different source sizes. An average visibility is calculated over a row of 14 pixels with 75 µm pixel. All the gratings were modeled according to Fig. S2 in the supplementary material.

In all non-destructive measurements of the X-LIGA gratings (Figs. 1 and 3), lower absorbing regions that may originate from improper gold filling of the polymeric template or gaps in it introduced by cracking and deformation were observed. To investigate the influence of missing gold filling in trenches, simulations were conducted on the G0 gratings with different source sizes and different numbers of missing trenches (simulated models are shown in the supplementary material in Fig. S2). Fig. 5a depicts simulated visibility profile on a detector row for a source size of 20 µm. The grating area that contributes positively to the interference pattern shrinks with the number of missing trenches, the latter being proportional to the reduction of visibility assessed at the affected pixel. In Fig. 5b, the visibility profile on a detector for a single missing trench is predicted for different source sizes, as the active area from G0 seen from a single detector pixel depends on the source size. With increasing source size, the gap in the visibility profile shrinks. However, the mean visibility around the missing trench on the detector decreases as the gap affects more pixels with larger source sizes than smaller ones.

Another artifact, reported in Ref.^3^, is local defects in the periodic structure of the gratings—which are referred to here as cracks - leading to local shifts. A single crack can cause drastic visibility reductions depending on the source size. These effects may occur when a grating is distorted due, for example, to improper bending to accommodate the cone beam angle. In Ref.^37^, a simulation of a local $\frac{4\pi }{5}$-shift in the grating’s periodic structure was conducted for various source sizes. An increase in source size led to a broader region of low visibility and significantly lower mean visibility if multiple cracks were present. An extension to the simulations conducted in Ref.^37^ can be seen in Fig. 5c where the visibility for different shifts in the period and source size are depicted. While the intensity remains unaffected by the shift, visibility—and consequently sensitivity - decreases with larger cracks and source sizes. This behavior differs from that caused by unfilled trenches. For empty trenches, the absolute visibility reduction is averaged over a larger detector area depending on the source size. In contrast, a crack results in the same absolute drop in visibility for any source size and extends the reduction of visibility to neighboring pixels, which reduces the mean visibility over a predefined area significantly.

The described defects were simulated for the G0 grating alone, as defects in the G2 grating cause only a local effect on a single pixel directly behind the grating, assuming the grating is positioned immediately in front of the detector. Consequently, missing trenches in G2 lead to a proportional reduction in visibility depending on the number of missing trenches, while a period shift results in a visibility drop with increasing shift, with maximum extinction occurring at odd multiples of $\pi$.

**Fig. 6:**
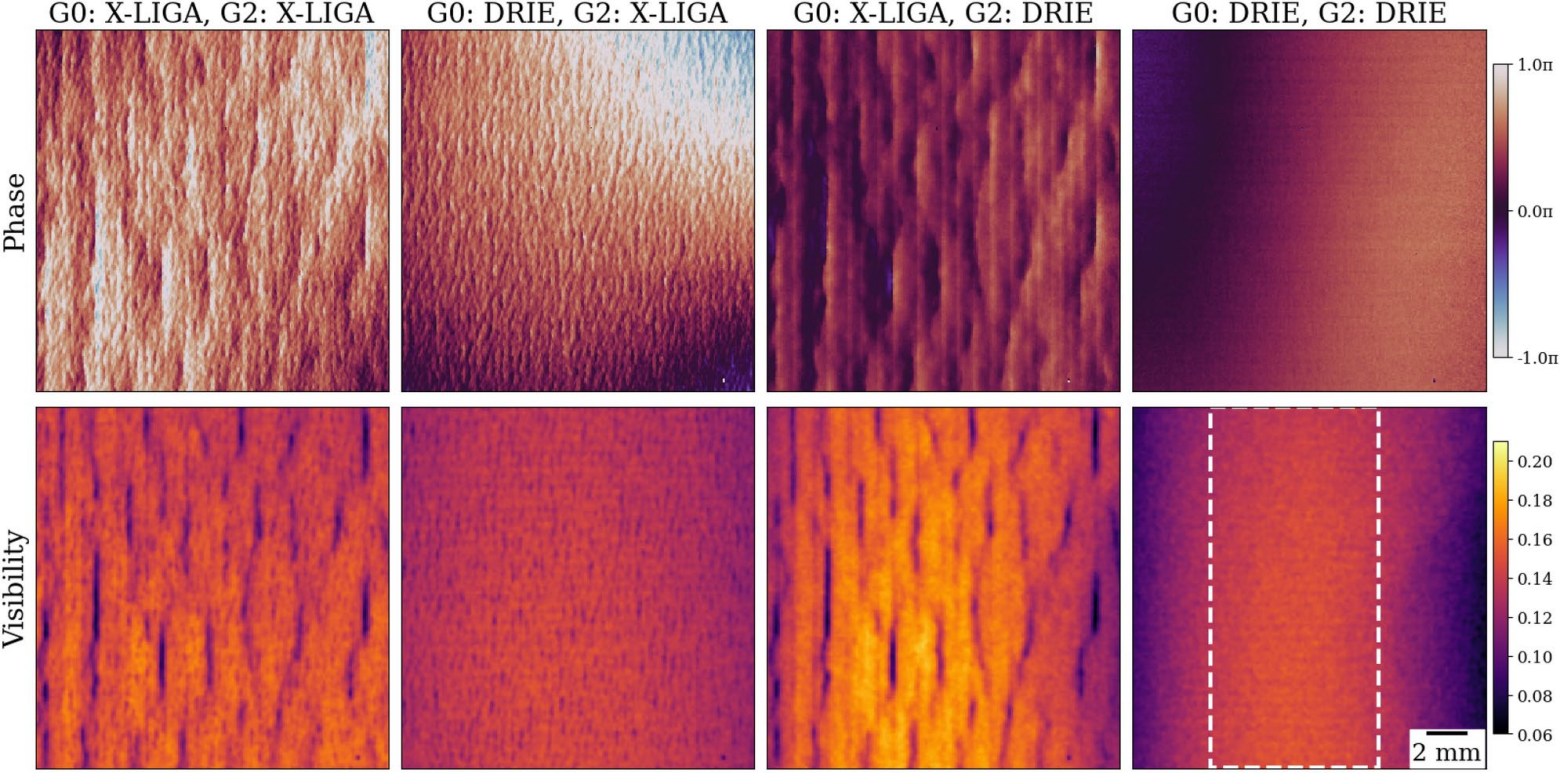
Phase and visibility maps of the grating pairs after signal retrieval using a least-squares fit to Eq. 4. Four pairs of gratings are analyzed: the X-LIGA grating used as G0 in the 1st configuration is used as well as G0 in the 3rd configuration, etc. In all the maps obtained with at least one X-LIGA grating present, visible discontinuities result in a higher heterogeneity in the noise of the images. In contrast, the DRIE gratings yield highly uniform maps for both phase and visibility. Albeit highly inhomogeneous, the highest local values of visibility are found with the combination of G0: X-LIGA, G2: DRIE. The scalebar shows the scale on the detector plane.

## Results

### Performance in a Talbot-Lau interferometer: experiment

**Fig. 7:**
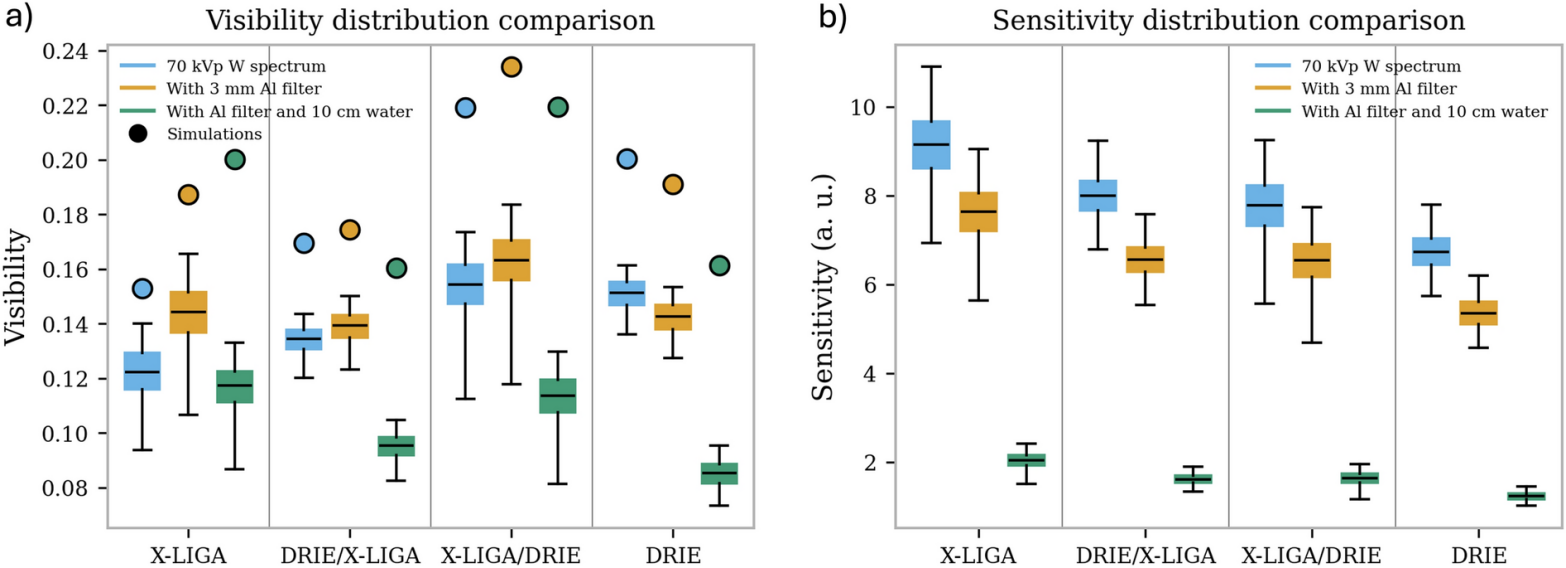
Visibility and sensitivity distribution for different spectra and different grating geometries on a region of interest (ROI) of 236 $\times$ 110 pixels. The quantiles shown span the 1 to 99 % range of the experimental data, with the colored boxes capturing the 25 to 75 % ranges, with the line in the center indicating the median value. (**a**) Visibility distribution from measurement is overlayed with the optimal values of the simulation. The visibility images were filtered first using a Gaussian filter with a kernel size of one pixel to reduce the influence of noise on the statistics. (**b**) Sensitivity calculated as the product of the $\sqrt{I}V$. Although the visibilities are similar, the reduction in transmission from the DRIE gratings leads to overall lower sensitivity.

A table-top XGI system was used to perform a pairwise comparison of two gratings, resulting in four different grating configurations being tested. The reason for the pairwise comparison is two-fold. Firstly, the two DRIE gratings differ in height and show different duty cycles. Secondly, the defects in the X-LIGA gratings affect the performance of an XGI differently, as effects from defects on G0 are more dependent on the source size than effects from defects on G2. The interpretation of the results and the simulations are addressed in the Discussion section.

The Moiré pattern seen by the detector was investigated by its phase and visibility (contrast). The phase, between $-\pi$ and $\pi$, is the relative position between the interference line pattern produced by G0 and G1 and the trenches of G2; it is zero if they perfectly overlap. Fig. 6 depicts the measured phase and visibility maps. The gratings were aligned to obtain a homogeneous phase map with the highest average visibility. Noticeable differences are evident in the uniformity of the phase maps between the X-LIGA and DRIE gratings. The DRIE gratings yield a smooth surface on the phase map, a crucial aspect for signal-retrieval with a sample since non-uniform phase maps are more difficult to correct for potential phase drifts and lead to higher heterogeneity in the noise of refraction images. In contrast, abrupt changes are seen in the phase map obtained using the X-LIGA gratings and found to correspond to lines of low values in the visibility map. Generally speaking, two scales of low-visibility regions are observed on the X-LIGA maps. The large regions come from the G0 X-LIGA grating, apparent when the G0 grating was replaced with the DRIE grating. The narrow visibility valleys originate from the G2 X-LIGA. The difference in size is due to the magnification of the cone-beam geometry. The DRIE gratings do not show any irregularities in either of the maps, as is expected from the absorption images (see Fig. 1).

Consistent with the use of flat gratings, only the central region of the detector area has been used for the quantitative analysis since rays propagating at a certain angle (which depends on the height and pitch of the gratings) will not lead to an interference pattern at the detector plane. A region with high and uniform visibility has been chosen from the DRIE measurements with the flat gratings for quantitative analysis (white boxed area). The X-LIGA gratings were used with their intrinsic bending radius of 300 mm, which increased the functional area on the detector but not the performance within the central region examined here.

In Fig. 7a, the visibility distribution of the individual measurements is shown for different X-ray spectra. The X-LIGA gratings show higher visibility in a spectrum filtered with 3 mm aluminum (hardened) than in the unfiltered spectrum. The DRIE gratings show the opposite behavior. However, the visibility difference with and without a filter is less prominent with the DRIE gratings than with the X-LIGA gratings. Where at least one X-LIGA grating is used, the filtered X-rays yield higher visibility due to the comparatively superior absorption of higher energy X-rays in the thicker Au. Combining an X-LIGA G0 and DRIE G2 is especially beneficial for the visibility, both with and without a filter. However, after a 10 cm water sample, the visibility is reduced when using either one of the DRIE gratings, while the X-LIGA gratings demonstrate only a relatively moderate drop in visibility. Another aspect is the width of the visibility distribution. When a DRIE grating is used as G0 the distribution is narrower, while a G0 X-LIGA gives a wide distribution and heterogeneity in the map.

The second important quantity for determining the sensitivity is the number of photons. In Table 1, the ratio of the absorption, i.e., the loss of intensity, compared to the configuration with only X-LIGA gratings is listed. As expected from the simulations, the DRIE gratings have higher absorption because the Au-filled features are trapezoidal, even if shallower, and reach almost a factor of three more absorption in the unfiltered spectrum.

With these two quantities, the sensitivities for the different configurations are calculated and summarized in Fig. 7b. In every case, the paired X-LIGA gratings provided the highest sensitivity. The X-LIGA gratings couple high visibility with higher overall transmission; the DRIE gratings achieve high visibility but suffer from higher absorption due to the trench tapering.

**Table 1:** Average values of absorption of different grating pairs obtained from the measurements and simulations scaled to the values obtained with G0 and G2 X-LIGA. Both the X-LIGA gratings have the same geometry and height (180 µm and 50 % duty-cycle). The DRIE gratings for G0 and G2 have different geometries, the latter with effectively thicker Au as already noted, explaining the difference in transmission when comparing DRIE/X-LIGA and X-LIGA/DRIE.

Ratio to X-LIGA	Unfiltered		3 mm Al filtered		3 mm Al + 100 mm H_2_O	
(G0 and G2)	Measurement	Simulation	Measurement	Simulation	Measurement	Simulation
DRIE / X-LIGA	1.57	1.85	1.26	1.44	1.06	1.22
X-LIGA / DRIE	2.19	2.17	1.74	1.74	1.45	1.49
DRIE	2.81	3.36	1.98	2.29	1.47	1.76

### Performance in a Talbot-Lau interferometer: simulation

Geometrical data obtained from SEM and optical imaging of the gratings was used to inform the model (see Methods section and Table 2 below). The accuracy of the model was then assessed by comparison of simulations with experimental results.

**Fig. 8:**
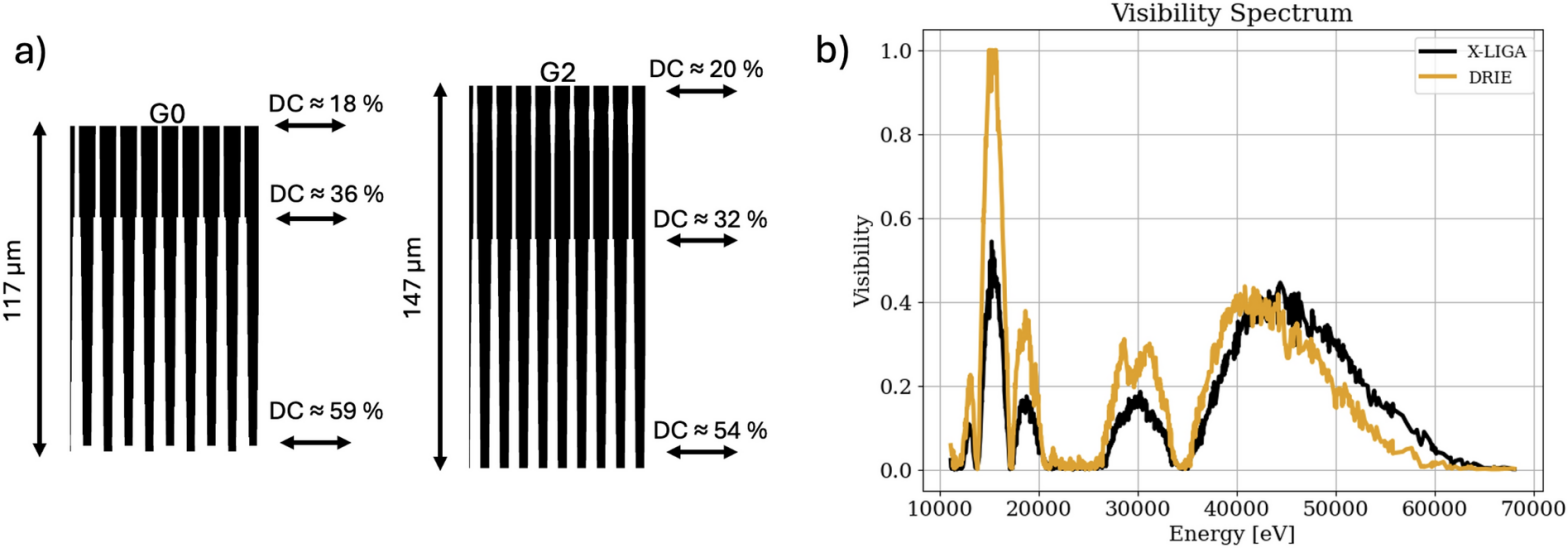
(**a**) Profile of the silicon template extracted from the SEM images for the DRIE gratings. (**b**) Visibility spectra for the pairs of DRIE and X-LIGA gratings. Because the DRIE gratings are shallower than the X-LIGA gratings, i.e., have less Au height for X-ray absorption, the visibility spectrum drops faster at higher energies. However, the lower mean duty cycle associated with their tapering allows for higher visibility at lower energies.

The simulation results are also included in Table 1 and Fig. 7. Although the DRIE gratings have shallower Au-filled trenches, they absorb the most incoming intensity in all of the configurations and illumination conditions examined. As per Table 1, absorption in the paired DRIE gratings is predicted to be 3.36$\times$ that with the paired X-LIGA gratings, reasonably consistent with the experimental observation. Fig. 7a compares the predicted visibilities with those measured experimentally. It is evident that the simulations capture, at least qualitatively, most of the trends observed experimentally. This includes the improved performance with hardened X-rays when at least one X-LIGA grating is used and the superior performance of the X-LIGA/DRIE grating combination for G0/G2. One obvious exception is the decrease of experimental visibility, where the model predicts an increase with the paired X-LIGA gratings in the presence of the water sample. Fig. 8b shows simulated visibility spectra for the DRIE and X-LIGA grating pairs. Due to the different duty cycles the visibility with the DRIE gratings is higher than with the X-LIGA gratings for energies up to 42 keV. However, the shallower Au-fill in the former leads to a lower visibility at higher energies and, consequently, a lower visibility for a harder spectrum.

## Discussion

### DRIE gratings

The uniformity of phase and visibility achieved with the DRIE gratings is exceptional and worthy of reiterating. Nonetheless, while the DRIE gratings provide high uniformity, they do not reach the same visibility as the X-LIGA gratings. Except for the configuration with X-LIGA as G0 and DRIE as G2, the visibility for pairings containing the DRIE grating(s) is reduced and significantly deteriorated when using a sample after G1. This is well understood given the relatively shallow Au fill in the DRIE gratings. Hardening of the X-ray spectrum, particularly with a specimen in the beam, causes a lower fringe contrast due to insufficient absorption from the Au-filled trenches of the DRIE grating, which is also seen in the simulated visibility spectrum in Fig. 8b. Although the Au-filled features are shallower, the overall absorption of the DRIE gratings is higher than the X-LIGA gratings, due to the lower mean duty cycle (approximately 31 %) of the tapered DRIE gratings versus the X-LIGA gratings (50 %). The combination of higher absorption and lower visibility leads to an overall lower sensitivity. Even though the combination with the X-LIGA G0 and DRIE G2 achieves similar visibility, it cannot reach the same sensitivity as the X-LIGA gratings. With a DRIE grating as G0, the exposure time could be increased for the same sample dose, potentially resulting in higher sensitivity. Modeling of the impact of tapering indicates sensitivity has an optimum at a rectangular-shaped form of 40 % duty cycle. For visibility, the peak is found for a grating with tapering, which differs from the solution found in Ref.^14^. Comparing the maximum visibility for a rectangular shape gratings against the maximum calculated visibility, the difference between those values is less than 1 % absolute (24.1 % vs. 24.3 % visibility). However, with the intensity considered, the optimum grating is rectangular, aligned with the results from Ref.^14^. Furthermore, if the duty cycle remains fixed, corresponding to moving perpendicular to the diagonal line in Fig. 4, varying the degree of tapering leads to only a moderate reduction in visibility compared to changes in the mean duty cycle. Additionally, the distribution closely follows the analytical prediction for decreasing duty cycle. If the grating height is insufficient to prevent the complete absorption of incoming photons, the visibility will eventually decrease after a certain duty cycle reduction. However, with a lower duty cycle or more extensive tapering artifact, the signal retrieval based on least-squares fitting with a sine curve may lead to overestimated visibility values as the phase stepping curve is not equally divided between transmitting and blocking regions. An adapted least-squares or conventional min-max visibility calculation might provide more accurate results, but with the caveat of requiring more phase steps to accurately predict the form of the phase stepping curve.

Although the DRIE gratings suffer from insufficient Au height, their defects are regular and predictable over the complete active area, leading to uniform phase and visibility maps. Significantly, essentially complete filling of the nearly 150 µm deep trenches (see G2) indicates thicker Au deposits may be expected with progress in DRIE.

### X-LIGA gratings

The X-LIGA gratings have substantial defects in all configurations, leading to broad regions with low visibility when used as G0. From the X-ray projections and CL images, these regions arise from poor gold filling of the polymeric template. It can be speculated that this kind of defect is generated during the development step of the X-ray exposed mold, which can be quite challenging for high aspect ratio structures since the chemical reactants must diffuse through the full grating height, and the byproducts need to be efficiently removed as well in order to achieve a uniformly well-developed template. Partial development results in limited diffusion of the Au electrolyte with a consequent non-uniform Au-filling of the polymeric template.

Quantitative predictions of the effect of missing or poorly filled trenches are provided in the simulations. A larger decrease in visibility generally arises from an area with more missing trenches (Fig. 5a). While a small source size gives a drop in visibility that is stronger and more local, with a larger source size the individual valleys merge to cover a broader area on the detector, consequently leading to a lower mean visibility. Additionally, the uniformity of the visibility map is corrupted by a collection of missing trenches and cracks (phase shift of the lamellae) spread heterogeneously over the detector area. Such defects will lead to a drop in visibility and large deviations in the phase map. In particular, local jumps in the phase map are observed for cracks.

### Simulations

The simulation predicts the function of the gratings in a controlled environment while also considering the grating geometry. The simulated transmission ratios exhibit some deviation from experimental results and overestimate the amount of absorption from the DRIE gratings in both scenarios with a DRIE G0. With the caveat that all values are scaled to those for the X-LIGA pair, it is seen in Table 1 that only the G0: X-LIGA and G2: DRIE combination exhibits the same transmission ratio in simulation and measurement. As accurate cross-sectional information is required, differences may originate from inaccurate representation of the gratings, especially the X-LIGA gratings. Modest changes in the duty cycle will greatly influence the measured, as well as predicted, transmission and visibility. The impact of tapering on the transmission signal is also significant. The modeled DRIE G0 grating has a height of 117 µm as compared to the 180 µm of the X-LIGA grating, which for Au at 46 keV corresponds to an absorption of only 87 % for the former versus 96 % for the latter. Thus, the DRIE G0 should be less absorbing based solely on the height difference. Predictions of higher absorption in the DRIE gratings are associated with the modeled taper, as changing the material of the transmitting trenches from Si to C_5_H_8_O_2_ solely contributes less than 1 % to the addition in absorption, and the substrate material change from Si to graphite causes less than 4 %.

The simulated visibility values, ignoring voided Au filling and broken or misshaped trenches in the gratings, provide an upper limit to the theoretically feasible visibility. Nevertheless, the predicted values follow the trends of the measurements, and one might logically expect the highest points in the experimental distributions to be less impacted by local defects. Only the simulated values with a 10 cm water container deviate substantially from the measurement trends involving an X-LIGA grating. One explanation is that the effective height and duty cycle of the X-LIGA gratings are lower than those modeled in the simulation. Such a difference would explain the discrepancy in the absorption ratios (see Table 1) and the increase in visibility with a water sample (see Fig. 7a). With lower Au height, the visibility distribution in Fig. 8b would decrease faster for higher energies. The harder spectrum with 10 cm of water would also lead to prediction of a more substantial decrease in visibility, as observed in the experiment, due to insufficient absorption at higher energies.

## Conclusions

The two grating manufacturing techniques, DRIE and X-LIGA, result in distinct characteristic defects that manifest in the image quality of an XGI system. The effect of related grating parameters on the sensitivity was observed experimentally and predicted quantitatively. Cross-sectional investigation of the geometry of the gratings by microscopy and laminography yielded crucial details for modeling that enabled broadly accurate predictions of the performance in an XGI. Additionally, computed laminography yielded the first cross-sectional information for an X-LIGA gold-filled grating reported in the literature. Overall, the following statements can be made from all measurements for HAR gratings manufactured by the two techniques: X-LIGA permits the manufacture of higher aspect ratio trenches with controlled duty cycles leading to rectangular-shaped lamellae. However, irregularities in the gold filling and shifts in the gratings structure yield irregular and heterogeneous visibility and phase maps with local decreases in visibility that worsen with larger source sizes. DRIE gratings do not presently reach trenches of the same depth and aspect ratio but demonstrate excellent uniformity in phase and visibility maps, which leads to good performance even with larger source sizes. The presence of significant tapering can result in increased absorption, decreasing the sensitivity. It is expected that these observations will assist manufacturers in prioritizing relevant geometrical factors for quality control during the manufacture of gratings, while XGI system designers can leverage these measurements and methods to establish tolerance requirements.

## Methods

### Radiography and computed laminography at TOMCAT

The radiography images of the X-LIGA and DRIE grating were obtained using a monochromatic beam with 45 keV and 4 s exposure time at the TOMCAT beamline at the Swiss Light Source. The effective pixel size is 0.375 µm. The CL measurement was conducted with a polychromatic beam having a mean energy of 66.5 keV, using the same 2-axis sample manipulator as in Ref.^38^ to obtain cross-sectional information of one X-LIGA grating. The measurement was performed with the rotation axis tilted by 30°, 6000 projections over 360°, with every projection having an exposure time of 300 ms, resulting in a total acquisition time of 30 min. The distance to the detector was set at 3 cm at a tilt angle of 0° and a pixel size of 0.65 µm. Reconstruction was performed using an adapted filtered back projection algorithm accounting for the tilting angle of the rotation axis^39^.

### Measurement system and geometry

**Fig. 9:**
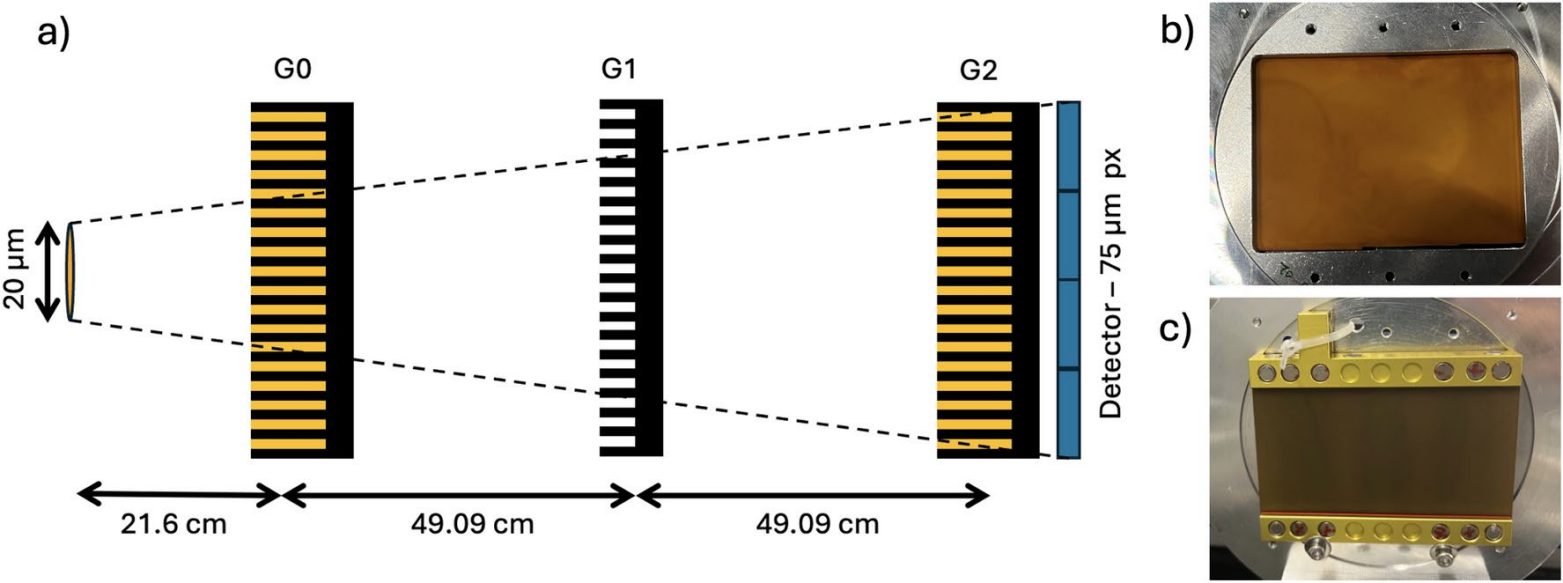
(**a**) Setup configuration of an XGI with all gratings mounted. The gratings were all mounted flat - except for G0 of X-LIGA, which was kept at its natural state of 30 cm bending radius. (**b**, **c**) DRIE and X-LIGA grating mounted on the holders, respectively. These gratings were used as G2.

The study was performed on a symmetric Talbot-Lau interferometer operated in the third Talbot order^12^ (see Fig. 9a). The gratings were mounted flat, except for the G0 grating from X-LIGA, which was used with its intrinsic 30 cm radius of curvature (see Fig. 9b,c for DRIE and X-LIGA G2 grating, respectively). The distance from the source to G0 was accordingly set to 21.8 cm to allow for the larger active area on G0 and larger high-visibility area on the detector plane to circumvent shadowing arising from the cone-beam of the source. The G0-to-G1 and G1-to-G2 distance at the 3rd Talbot order is 49.1 cm with a G1 grating designed to introduce a phase shift of $\pi$ at a design energy of 46 keV. The detector was placed directly after G2. The detector is a prototype photon counting detector by DECTRIS Ltd. (Baden-Daettwil, CH) with a 750 µm-thick Cadmium-Telluride layer as the active medium and an active area of 256 $\times$ 3098 isotropic 75 µm pixels. A single energy threshold of 11 keV was used for all measurements with a frame rate of 0.5 Hz. The X-ray source was a Hamamatsu L10101 microfocal tube operated at 70 kVp and 200 µA, with a focal spot size of 20 µm.

The signal retrieval is performed with a linear least-squares fit^40^ to every *i*-th pixel via:

4 $$\begin{aligned} I_{i, x} = I_i \cdot \left[ 1 + V_i \cdot \cos \left( \frac{2 \pi x}{p_{G2}} + \phi _i \right) \right] \end{aligned}$$

where $I_{i, x}$ is the intensity measured at the position *x* of G2. $I_i$ is the mean intensity, $V_i$ the visibility and $\phi _i$ the phase-shift of the phase stepping curve. For all experiments and simulations five phase-steps were conducted with G2.

In general, Eq. 4 depends on the X-ray spectrum^41^. However, the energy and weighting dependence are omitted for brevity. To see the influence of different spectra on the performance of the interferometer with different gratings, three different measurements were performed with: the source spectrum, the source spectrum filtered by 3 mm aluminum, and the source spectrum filtered by the aluminum plus a 10 cm water container directly after G1 to emulate a biological sample.

### Simulation parameters for measurement comparison

Using images such as those in Fig. 2b, the trench geometry of the DRIE gratings (see Fig. 8a) was modeled, while purely rectangular gratings were assumed for X-LIGA. The models assume a regular pattern across the entire grating area. However, as seen, the X-LIGA gratings, in particular, contain complex and irregular defects that affect the overall sensitivity. To assess the impact of individual features, the gratings were modeled as coherent, repeating structures, providing an upper level on the visibility of the system. Although typical photoresist contains a polymer, sensitizer and solvent, the solvent and sensitizer were assumed to have a negligible effect on X-ray absorption so only the polymeric component was considered in simulations of the X-LIGA gratings. A composition of C_5_H_8_O_2_ was used for convenience, but it can be replaced with specific formulation of typical X-ray photoresists, such as polymethylmethacrylate (PMMA) or epoxy-based SU8, with a negligible effect on the final spectrum.

For X-LIGA, the gratings were modeled with a height of 180 µm and a duty cycle of 50 %. The trench material was modeled as PMMA and silicon for X-LIGA and DRIE, respectively. As substrate material, graphite was used for X-LIGA and silicon for DRIE. The G1 grating has a height of 59 µm and the trenches and substrate are modeled as silicon. All absorption gratings were modeled to have a solid gold filling. Individual spectra were modeled using the python package SpekPy^42^, for a 70 kVp tungsten source with a 0.15 mm Be window. For each simulation, 1000 individual source points following the spectrum distribution and uniformly randomly distributed on a source size of 20 µm were sampled, according to Ref.^37^. The intensity profile of each source point was also finally weighted by the detector response curve.

**Table 2:** Grating parameters extracted from SEM and optical imaging. These parameters were used for the pairwise comparison of gratings, as summarized in Fig. 7 and Table 1. Note that the duty cycle for the DRIE gratings was modeled according to Fig. 8a, and the height in brackets represents the total grating height, including voids in the gold filling. For the tapered grating study, the DRIE gratings had a height of 140 µm.

X-LIGA							
	Height	Pitch	$\hbox {DC}_{{top}}$	$\hbox {DC}_{bottom}$	Trench Material	Substrate Material	Substrate height
G0	180 µm	4.2 µm	50 %	50 %	C_5_H_8_O_2_	Graphite	320 µm
G2	180 µm	4.2 µm	50 %	50 %	C_5_H_8_O_2_	Graphite	320 µm

DRIE							
G0	117 µm (145 µm)	4.2 µm	18 %	59 %	Silicon	Silicon	253 µm
G2	147 µm (152 µm)	4.2 µm	20 %	54 %	Silicon	Silicon	223 µm

## Supplementary Information

Supplementary Information.

## Data Availability

The datasets analyzed during the current study are available on ETH Research Collection (https://doi.org/10.3929/ethz-b-000724659), and the code for the analysis will be published on GitHub (https://github.com/eth-xrm/grating_defects).
