# Supplementary material for: Quantifying grating defects in X-ray Talbot-Lau interferometry through a comparative study of two fabrication techniques
Source: Sci Rep. 2025 Apr 24;15:14223. doi: 10.1038/s41598-025-98148-z (PMC12022164; doi:10.1038/s41598-025-98148-z)
Supplement: Supplementary file 1 — Supplementary Information. [file 41598_2025_98148_MOESM1_ESM.pdf]

Supplementary Material

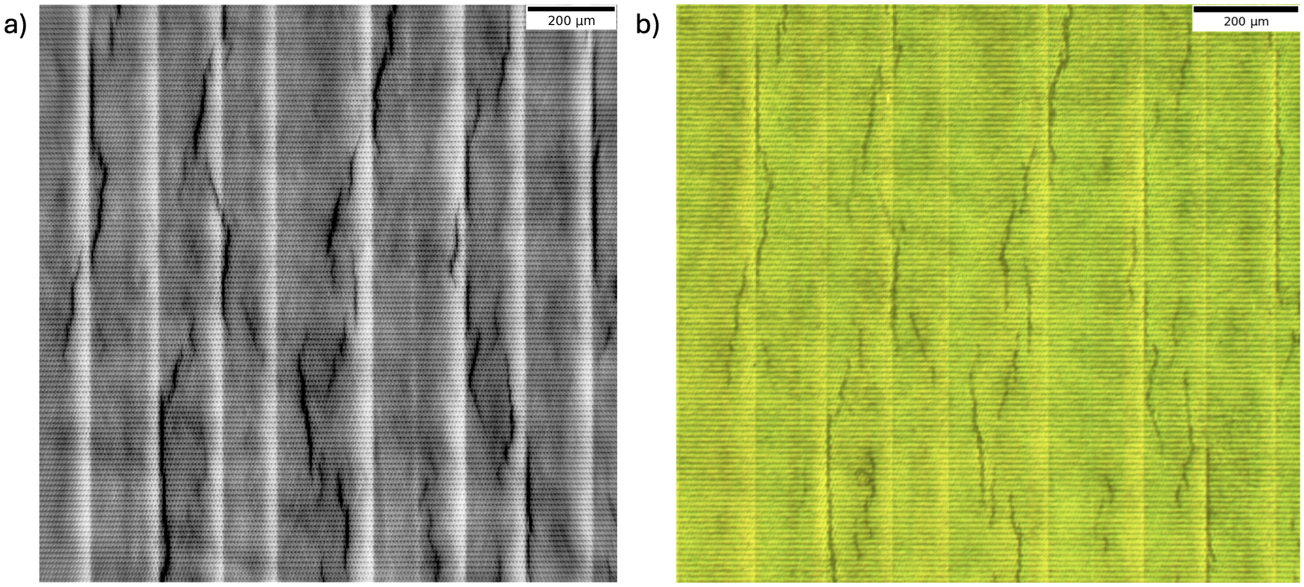

Figure S-1. a) Slice of the laminography scan matched to an b) optical microscope image. The slice of the laminography corresponds to the same slice as in Fig. 3 of the main article. Here it becomes apparent that the highly transmissive areas propagate through the full thickness of the grating, as the optical microscope image shows only the surface of the grating.

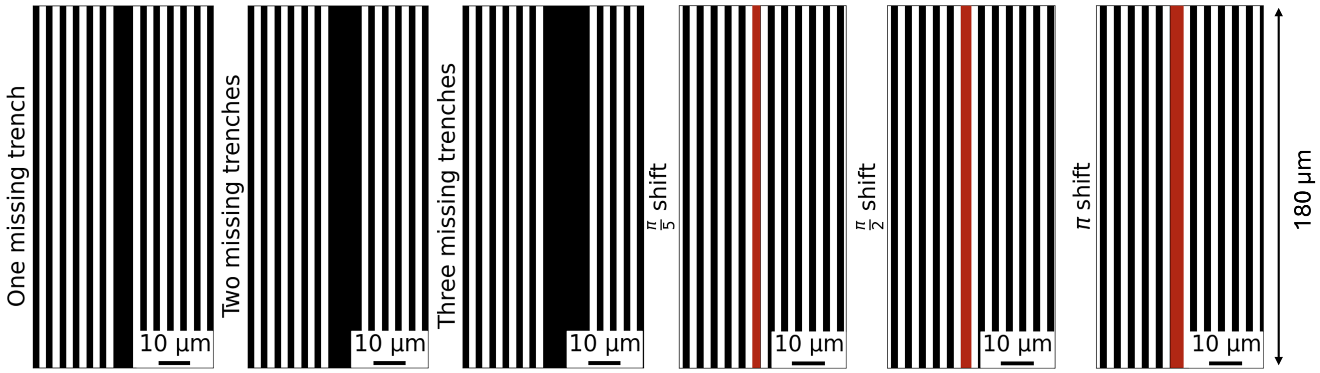

Figure S-2. Models of the gratings used to simulate defects in X-LIGA gratings. The three left images depict missing trenches, corresponding to Fig. 5a and 5b. The three right images illustrate local period shifts, used for Fig. 5c, with shifts marked in red for clarity.
